# COVID-19 infection and vaccine have no impact on in-vitro fertilization (IVF) outcome

**DOI:** 10.1038/s41598-022-25757-3

**Published:** 2022-12-15

**Authors:** Soha Albeitawi, Zina M. Al-Alami, Jehan Hamadneh, Hiba Alqam, Hussein Qublan, Maha Al Natsheh

**Affiliations:** 1 Clinical Science Department, Faculty of Medicine, Yarmouk University, P.O.Box: 566, Irbid, 21163 Jordan; 2 Faculty of Allied Medical Sciences, Al-Ahliyya Amman University, Amman, Jordan; 3 Obstetrics and Gynecology Department, Jordan University of Science and Technology, Irbid, Jordan; 4 Irbid Specialty Hospital IVF Center, Irbid Specialty Hospital, Irbid, Jordan; 5 Al Kindi IVF Unit, Al Kindi Hospital, Amman, Jordan

**Keywords:** Patient education, Outcomes research

## Abstract

To investigate the effect of COVID-19 infection or vaccine on IVF outcome. This is a multicenter retrospective study. Data were collected from all patients treated in the ART units between September and November 2021 after the vaccination of the general population began. Medical records of all patients who had IVF/intracytoplasmic sperm injection (ICSI) were retrospectively reviewed. Patients were categorized into four groups: previously infected by COVID-19, vaccinated by COVID vaccine, previously infected and vaccinated, or neither infected nor vaccinated. Total number of participants 151 (vaccinated only 66, infected only 18, vaccinated and previously infected 34, and control 33. Outcomes (ET on day of trigger, number of oocytes retrieved, quality of oocytes, number of fertilized oocytes, number and quality of embryos, number of embryos transferred, number of embryos frozen, implantation rate and clinical pregnancy rate) were compared between these four groups. Moreover, we compared the outcome before and post infection, as well as before and post vaccine in a group of patients. No evidence was found to suggest that COVID-19 disease or SARS-CoV-2 Vaccine adversely affects Clinical pregnancy rates (positive fetal heartbeat) (OR 0.9, CI 0.5–1.9, OR 1.8, CI 0.9–3.6, respectively) and the following parameters: fertilization rate, implantation rate, positive bHcg) (OR 0.9, CI 0.5–1.8, OR 1.5, CI 0.7–2.9, respectively). Although a limitation of our study is the small comparison groups, and the wide confidence intervals in the Odds Ratio estimates.

## Introduction

COVID-19 disease, caused by coronavirus SARS-CoV-2 was declared by World Health Organization as pandemic on 11th March 2020^1,2^. More than 100 million cases have been recognized worldwide, and over 2.5 million people have died due to the disease^3^. Fever, headache, myalgia, cough, shortness of breath, diarrhea, and anosmia are the most common symptoms of COVID-19^4^. SARS-CoV-2 enters into target host cells via the angiotensin converting enzyme 2 (ACE2), and needs cellular protease such as transmembrane protease serine (TMPRSS)^5^. Therefore, theoretically, organs with a high expression of ACE2 and TMPRSS2 are more vulnerable to infection. Results of immunohistochemistry and single-cell RNA sequencing data have indicated that there is high expression of ACE2 in the testis, and in the ovaries^6–9^. Therefore, the ovary is potentially vulnerable to SARS-CoV-2 infection. Furthermore, ACE 2 is expressed and Angiotensin- (1–7) Mas receptor-ACE2 axis is functioning in all stages of follicular maturation in the human ovary^9^. In addition, the rate of oocyte maturation is found to be related to the level of Angiotensin (1–7) in the follicular fluid^10^.

However, SARS- CoV-2 viral particles hasn’t been detected yet in the ovaries^11–14^. Yaakov Bentov et al. analyzed serum and follicular fluid for anti-COVID IgG, estrogen, and progesterone concentration, as well as the number and maturity of aspirated oocytes and previous estrogen and progesterone measurements. They found that both COVID-19 infection and vaccination with the BNT162b2 mRNA vaccine have no detrimental effect on follicular function^15^.

ACE2 has been detected as well in early embryos before the 8- cell stage in addition to the trophectoderm cells of late blastocysts, and TMPRSS2 is present in the late stage blastocyst, therefore peri-implantation embryos are highly susceptible to SARS-COV-2 infection^16,17^. Wang et al. analyzed assisted reproductive technology data and they found that SARS- CoV-2 infection didn’t affect female fertility and embryo development^18^. While, Raoul Ovrieto et al. found that couples infected with COVID-19 had lower proportion of top quality embryos but no impact was observed on patient’s performance and ovarian reserve as well. It worth to mention that the number included in this study was only 9 couples and in two of them the male partner who was infected rather than the female^19^. Furthermore, Yamila Herrero et al. compared the ovarian function of 34 patients who had never been infected versus 46 who had recovered from COVID-19. They found that patients with higher IgG levels in the follicular fluid had fewer retrieved oocytes. The authors of the study concluded that COVID-19 infection negatively affects the follicular microenvironment. It should be noted that the effect of this infection on the clinical outcome of IVF cycle was not investigated in this study. In addition, the study does not answer the question of how long does this effect last^20^.

ACE2 is present in the endometrium and the expression varies with the menstrual cycle phase, being stronger during the secretory phase^3,22^. The ACE2 has a vital role in endometrial proliferation and renewal. Accordingly, it is expected that the downregulation of ACE2 by SARS-CoV-2 could affect the endometrial stability and may impair implantation^22–26^. Whether this has a noxious effect on the endometrium due to COVID-19 infection or vaccination need to be clarified^27^. It has been reported that SARS-CoV-2 infection can cause transient menstrual cycle changes^28^. Another study found that the severity of viral infection is negatively associated with AMH Anti-Mullerian Hormone level^29^. Therefore, COVID-19 may cause ovarian injury and detrimentally affect ovarian reserve^21^.

In relation to COVID-19 vaccines, three main types are available in Jordan: mRNA vaccines, replication-defective live viral vectors based vaccines and virus inactivated vaccines^30^. The mRNA vaccine, BNT162b2, a Pfizer BionTech is the main vaccine that had been investigated. Myriam Safari et al. found that Pfizer BioNTech (BNT162b2) vaccine has no effect on intracytoplasmic sperm injection (ICSI) cycles outcome^31^. Their finding was confirmed by Raoul Orvieto et al.^32^. Moreover, it has been suggested that COVID-19 infection is unlikely to have long term effect on female reproductive tract however it need to be confirmed^33^. Recently, it has been published that SARS-Cov-2 spike protein sero-positivity from infection or vaccination does not prevent embryo implantation or early development^34^. Bowman and his team studied the effect on BNT162b2 vaccine on female fertility in rats. They found that it has no impact on fertility, ovarian or uterine parameters and embryo-fetal development^26^. Moreover, another study by Devora Ahron et al., they compared early IVF outcomes between 28 patients who received Pfizer vaccine, 37 patients received the Moderna vaccine and 328 unvaccinated patients who were a control group. They concluded that there was no association between COVID-19 vaccination and clinical pregnancy or current pregnancies^35^. Similarly, another study concluded that COVID-19 mRNA vaccine has no effect on ovarian response or pregnancy rate in patients who received the vaccine before IVF^36^. Furthermore, a very recent study showed that the number of retrieved oocyte, good quality embryos and percentage of clinical pregnancy rate were similar between 146 patients who received the inactivated SARS-CoV-2 vaccine (Sinopharm COVID-19 (BBIBP-CorV, COVILO) and in the 584 patients in the control group^37^.

In this study we set out to investigate whether COVID-19 infection or different types of vaccines(Pfizer(BioNTech), Oxford/AstraZeneca (ChAdOx1-S recombinant] vaccine) and/or Sinopharm (BBIBP-CorV)) affect on in-vitro fertilization (IVF) outcomes.

## Methods

### Data collection

This study is a multicenter retrospective study, that was carried out at 2 assisted reproduction technology (ART) centers, in Jordan, Al Kindi IVF center in Amman and Irbid Specialty Hospital IVF center in Irbid. Data were collected from a convenient sample of patients who visited the units between September and November 2021, after the vaccination of the general population began in January 2021. Medical records of patients who underwent IVF/intracytoplasmic sperm injection (ICSI) were retrospectively reviewed. Patients were divided into four groups: those previously infected with COVID-19, vaccinated against the disease, previously infected and vaccinated, or neither infected nor vaccinated. Total number of participants 151 (vaccinated only 66, infected only 18, vaccinated and previously infected 34, and control 33. The data obtained in the groups were compared. The following parameters were included: patient demographics (age); number of previous IVF/ICSI cycles, duration of infertility, causes of infertility, protocol used, injections used, numbers days of stimulation, endometrial thickness at day of triggering, triggering method used, number of retrieved oocytes, number of MII oocytes, ICSI versus conventional IVF, number of oocytes fertilized, number and grade of day 3 embryos, number and grade of blastocysts, day of transfer, number of embryos transferred, number of embryos/blastocyst frozen, positive pregnancy test, and presence of OHSS. The embryos’ quality at day 3 was determined by cell number, symmetry and fragmentation according to the Society for Assisted Reproductive Technology (SART) grading guidelines, grading was good, fair or poor. In addition, the fact and the time of previous infection with COVID-19 and the history of vaccination against coronavirus were recorded. Specifically, the type of vaccine, timing, and number of doses received prior to the cycle were documneted. The type of vaccine could include one of the three vaccines available in Jordan, namely Pfizer(BioNTech), Oxford-Astrazeneca (ChAdOx1-S recombinant) and/or Sinopharm (BBIBP-CorV COVILO). The primary outcomes, which were compared between the four groups, included: fertilization rate, implantation rate and clinical pregnancy rate. Secondary outcomes included: number of oocytes retrieved; number of mature oocytes; and the number and quality of embryos at day 3. The IVF outcomes in a group of 50 patients who underwent IVF cycle before and after the pandemic, were also compared.

### IVF protocol

Several protocols were used for controlled ovarian stimulation, short agonist protocol, flexible GnRH antagonist protocol and long agonist protocol. The starting dose of gonadotropin was decided according to patient’s age, ovarian reserve and BMI. Ovarian response was monitored by transvaginal ultrasound and gonadotropin dose changed accordingly. Once two leading follicles reaches a diameter of 17–18 mm or a dominant follicle 20 mm final oocyte maturation was triggered by human chorionic gonadotropin (HCG). Oocyte pickup was performed 35–37 h later. The oocytes were incubated for 2 h before the ICSI. The cumulus and corona cells were removed using enzymatic digestion by cumulase, in addition to utilizing denuding pipette for mechanical denudation. After 16 ± 2 h fertilization was assessed by looking for the 2 pronulcei (PN).

Luteal phase was supported by vaginal progesterone ± oral dydrogesterone 20 mg per day, started one day after pick up. Serum BhCG was measured 14 days after embryo transfer and value above 5 IU/ml was consider positive. Luteal phase support was continued until 10th week gestation. Embryo transfer was determined according to number and quality of embryos and the risk of ovarian hyper-stimulation syndrome.

### Statistical analysis

All extracted data were summarized in a Microsoft Excel workbook and analyzed using Statistical Package for Social Sciences (SPSS) version 23. The study’s results were reported in the form of descriptive statistics. The study’s results were reported in the form of descriptive statistics. Categorical variables were summarized in the form of frequencies [n (%)], while continuous data were reported as means, medians (when applicable) and standard deviations. Categorical associations were evaluated using Chi-square test, while associations involving continuous data were assessed using Student’s *t*-test. Because the data failed to be normally distributed, nonparametric tests such as Kruskal–Wallis test were utilized to assess the study’s hypotheses and detect significant differences. Paired parameters were tested using Wilcoxon paired test. An alpha value of ≤ 0.05 (CI = 95%) was considered statistically significant.

### Outcome measures and definitions

Fertilization rate is defined as the percentage of fertilized oocytes from the collected oocytes. Implantation rate is calculated as the number of gestational sacs observed per number of embryos transferred.

### Ethical approval

Institutional Review Board approval was obtained from the Ethical Reviewing Board at the Faculty of Pharmacy and the Faculty of allied medical sciences at Al-Ahliyya Amman University. Approval number: (AAU/11/5/2020–2021). All research was performed in accordance with relevant guidelines/regulations, and in accordance with the declaration of Helsinki. Since it is a retrospective review of medical records, the informed consent was waived by the Ethical Reviewing Board at the Faculty of Pharmacy and the Faculty of allied medical sciences at Al-Ahliyya Amman University.

## Results

### General characteristics

Total number of cases included in this study was 151. Table 1 demonstrates general characteristics of the patients.

**Table 1:** Patients’ characteristics.

Parameter	Parameter representation	After vaccine	After infection	Patients after vaccination and infection	Patients neither vaccinated nor infected	Total	P value
Number	N	66	18	34	33	151	
Age	Mean ± SD	35.56 ± 6.14	31.44 ± 6.22	33.47 ± 5.43	30.93 ± 7.42	33.58 ± 6.54	0.003
Duration of infertility (years or months?)	Mean + -SD	8.12 ± 5.06	5.66 ± 3.06	6.00 ± 3.25	5.34 ± 3.86	6.75 ± 4.38	0.013
Indication n (%)	Female factor	22 (33.3)	4 (22.2)	13 (38.2)	10 (30.3)	49 (32.5)	0.263
Indication n (%)	Male factor	23 (34.8)	3 (16.7)	8 (23.5)	12 (36.4)	46 (30.5)	Non applicable?
Indication n (%)	Combined	7 (10.6)	1 (5.6)	1 (2.9)	2 (6.1)	11 (7.3)	
Indication n (%)	Idiopathic	4 (6.1)	0.00	2 (5.9)	4 (12.1)	10 (6.6)	
Indication n (%)	Unexplained	3 (4.5)	4 (22.2)	4 (11.8)	1 (3.0)	12 (7.9)	
Indication n (%)	PGD	6 (9.1)	4 (22.2)	4 (11.8)	4 (12.1)	18 (11.9)	
Number of Previous IVF cycle	Mean ± SD	1.64 ± 2.45	1.44 ± 1.72	0.94 ± 1.25	0.78 ± 1.29	1.27 ± 1.94	0.132

*SD* standard deviation, *PGD* preimplantation genetic diagnosis.

### IVF parameters

Tables 2 and 3 show the difference in the results of IVF parameters among different groups of participants.

**Table 2:** COVID-19 and IVF parameters outcome.

	Parameter Rep	Have been infected (n = 52)	Were not infected (n = 98)	Mean difference	Lower 95% CI	Upper 95% CI	p-value
Numbers of days of stimulation	Mean ± SD	8.9 ± 2.0	9.6 ± 1.9	− 0.59	− 1.24	0.06	0.07
Number of oocytes retrieved	Mean ± SD	12.1 ± 10.6	11.2 ± 8.4	0.91	− 2.22	4.03	0.56
Number of MII oocytes retrieved	Mean ± SD	9.5 ± 7.6	9.1 ± 6.5	0.43	− 1.91	2.77	0.71
Number of fertilized oocytes	Mean ± SD	8.1 ± 6.4	7.2 ± 5.5	0.90	− 1.06	2.87	0.36
Number of cleavage stage embryos (D2 or D3)	Mean ± SD	5.4 ± 4.9	5.6 ± 4.5	− 0.17	− 1.75	1.40	0.82
Number of embryos transferred	Mean ± SD	2.5 ± 1.3	2.1 ± 1.3	0.37	− 0.06	− 0.8	0.09
Fertilization rate^a^	Mean ± SD	74.1 ± 21.5	68.1 ± 23.1	5.98	− 1.66	13.6	0.12
Implantation rate^b^	Mean ± SD	48.3 ± 22.1	59.0 ± 27.4	− 10.7	− 25.4	3.99	0.15

	Parameter Rep	Have been infected (n = 52)	Were not infected (n = 98)	OR	Lower 95% CI	Upper 95% CI	p-value
Trigger used	DT	3 (5.8)	12 (12.1)	0.44	0.11	1.65	0.21
	HCG only	49 (94.2)	87 (87.9)				
Grade of cleaved embryo	Grade 1	46 (90.2)	81 (91.0)	0.90	0.28	2.94	0.87
	Grade 2 or 3	5 (9.8)	8 (9.0)				
Day of transfer	2 – 3	4 (7.7)	18 (18.2)	0.37	0.12	1.17	0.09
	4 – 5	48 (92.3)	81 (81.8)				
Pregnancy test + (BhCg)	Negative	31 (59.6)	61 (61.6)	0.92	0.46	1.82	0.86
	Positive	21 (40.4)	38 (38.4)				
Pregnancy + (FHB)	Negative	31 (62.0)	61 (63.5)	0.93	0.46	1.89	0.85
	Positive	19 (38.0)	35 (36.5)				
OHSS	Negative	48 (92.3)	93 (95.9)	0.51	0.12	2.15	0.45
	Positive	4 (7.7)	4 (4.1)				

*SD* standard deviation, *DT* dual trigger, *BhCG* beta human chorionic gonadotropin, *HCG* human chorionic gonadotropin, *OHSS* ovarian hyper-stimulation syndrome, *OR* Odds ratio, *CI* Confidence interval.

^a^Fertilization rate is defined as the percentage of fertilized oocytes from the collected oocytes. ^b^Implantation rate is calculated as the number of gestational sacs observed per number of embryos transferred.

**Table 3:** Vaccine and IVF treatment outcome parameters.

	Parameter Rep	Vaccinated (n = 100)	Non vaccinated (n = 51)	Mean difference	Lower 95% CI	Upper 95% CI	p-value
Numbers of days of stimulation	Mean ± SD	9.3 ± 1.8	9.5 ± 2.2	− 0.15	− 0.82	0.51	0.64
Number of oocytes retrieved	Mean ± SD	10.6 ± 8.4	13.2 ± 10.6	− 2.61	− 5.73	0.50	0.10
Number of MII oocytes retrieved	Mean ± SD	8.5 ± 6.0	10.6 ± 8.2	− 2.18	− 4.51	0.14	0.06
Number of fertilized oocytes	Mean ± SD	6.8 ± 4.9	8.7 ± 7.2	− 1.90	− 3.86	0.05	0.05
Number of cleavage stage embryos (D2 or D3)	Mean ± SD	4.8 ± 3.7	6.8 ± 5.9	− 1.97	− 3.53	− 0.41	**0.01**
Number of embryos transferred	Mean ± SD	2.3 ± 1.3	2.1 ± 1.2	0.23	− 0.21	0.68	0.30
Fertilization rate^a^	Mean ± SD	70.3 ± 23.0	69.8 ± 22.3	0.53	− 7.21	8.27	0.89
Implantation rate^b^	Mean ± SD	51.2 ± 22.7	60.8 ± 29.4	− 9.74	− 23.9	4.59	0.17

	Parameter Rep	Vaccinated (n = 100)	Non vaccinated (n = 51)	OR	Lower 95% CI	Upper 95% CI	p-value
Trigger used	DT	8 (8.0)	7 (13.7)	0.54	0.186	1.60	0.26
	HCG only	92 (92.0)	44 (86.3)				
Grade of cleaved embryo	Grade 1	80 (86.0)	47 (100.0)	NA	NA	NA	NA
	Grade 2 or 3	13 (14.0)	0 (0.0)				
Day of transfer	2–3	15 (15.0)	7 (13.7)	1.10	0.42	2.92	0.83
	4–5	85 (85.0)	44 (86.3)				
Pregnancy test + (BhCg)	Negative	64 (64.0)	28 (54.9)	1.46	0.73	2.9	0.29
	Positive	36 (36.0)	23 (45.1)				
Pregnancy + (FHB)	Negative	65 (67.7)	27 (54.0)	1.78	0.88	3.60	0.10
	Positive	31 (32.3)	23 (46.0)				
OHSS	Negative	93 (94.9)	48 (94.1)	1.16	0.26	5.07	0.84
	Positive	5 (5.1)	3 (5.9)				

*SD* standard deviation, *DT* dual trigger, *BhCG* beta human chorionic gonadotropin, *HCG* human chorionic gonadotropin, *OHSS* ovarian hyper-stimulation syndrome, *OR* Odds ratio, *CI* Confidence interval.

^a^Fertilization rate is defined as the percentage of fertilized oocytes from the collected oocytes. ^b^Implantation rate is calculated as the number of gestational sacs observed per number of embryos transferred.

### Previous COVID-19 infection

Our findings indicate that previous COVID-19 infection does not affect any of the following IVF outcomes including fertilization rate, implantation rate. Similarly there was no difference in the pregnancy rate as determined by positive BHcg or clinical pregnancy (OR 0.92, CI 0.463–1.827, OR 0.936, CI 0.462–1.897) respectively. Additionally, IVF parameters didn’t have a significant difference between those who had COVID-19 or not Table 2.

Moreover, the mean number of retrieved oocytes and the number, as well as class of embryos did not differ significantly before and after the COVID-19 infection (Table 4).

**Table 4:** IVF parameters outcome before and after vaccine/infection.

Parameter	Vaccinated before	Vaccinated after	p value	Infected before	Infected after	p value	Both before	Both after	p value	All before	All after	p value
Numbers of days of stimulation	7.5 ± 1.2	7.7 ± 1.3	0.186	7.22 ± 1.09	7.55 ± 2.55	0.785	7.86 ± 1.84	7.86 ± 1.59	0.999	7.6 ± 1.4	7.8 ± 1.8	0.742
HCG trigger n (%)	23 (100)	23 (100)		9 (100)	9 (100)		15 (100)	15 (100)		51 (100)	51 (100)	–
Number of oocyte retrieved	8.3 ± 4.6	8.1 ± 7.1	0.425	12.44 ± 6.48	12.00 ± 7.05	0.514	11.40 ± 7.86	10.86 ± 7.38	0.431	10.1 ± 6.1	9.6 ± 7.0	0.124
Number of MII oocytes retrieved	6.6 ± 3.8	6.5 ± 5.3	0.613	10.00 ± 4.89	9.22 ± 5.84	0.440	7.80 ± 4.85	8.00 ± 3.83	0.782	7.5 ± 4.3	7.4 ± 4.9	0.649
Number of fertilized oocytes	5.6 ± 3.1	5.8 ± 4.6	0.806	9.11 ± 4.85	8.33 ± 5.59	0.171	6.80 ± 4.19	7.73 ± 4.04	0.502	6.57 ± 3.83	6.85 ± 4.57	0.726
Number of cleavage stage embryos (D2 or D3)	4.0 ± 2.2	2.7 ± 2.2	0.011	7.33 ± 4.44	6.00 ± 5.65	0.154	4.40 ± 1.80	4.40 ± 2.99	0.774	4.6 ± 2.8	3.9 ± 3.4	0.015
Grade 1 (D2 or D3 embryos)	18 (78.3)	21 (91.3)	–	8 (88.9)	9 (100)	–	14 (93.3)	9 (60.0)	–	43 (84.3)	43 (84.3)	–
Grade 2 or 3 (D2 or D3 embryos)	5 (21.7)	2 (8.7)	–	1 (11.1)	0 (0.0)	–	1 (6.7)	6 (40.0)	–	8 (15.7)	8 (15.7)	–
Number of day 5 embryos (blastocyst)	0.0	0.13 ± 0.62	0.317	1.22 ± 2.53	1.00 ± 3.00	0.655	0.26 ± 1.03	0.06 ± 0.25	0.655	0.3 ± 1.2	0.2 ± 1.3	0.684
Day 2–3 of transfer	20 (87.0)	10 (43.5)	0.024	7 (77.8)	6 (66.7)	0.999	9 (60.0)	7 (46.7)	0.317	39 (76.5)	27 (52.9)	0.039
Day 4–5 of transfer	1 (4.3)	7 (30.4)		2 (22.2)	3 (33.3)		5 (33.3)	8 (53.3)		9 (17.6)	18 (35.3)	
Number of embryos transferred	2.56 ± 1.03	1.78 ± 1.34	0.008	3.00 ± 0.00	2.66 ± 0.86	0.257	2.46 ± 1.06	2.26 ± 0.59	0.559	2.6 ± 0.9	2.1 ± 1.1	0.006
Number of positive pregnancy test	14 (60.9)	8 (34.8)	0.058	4 (44.4)	6 (66.7)	0.317	10 (66.7)	6 (40.0)	0.102	30 (58.8)	20 (39.2)	0.052
Number of clinical pregnancy (+ fetal heart beat)	12 (52.2)	6 (26.1)	0.058	3 (33.3)	3 (33.3)	0.999	7 (46.7)	6 (40.0)	0.655	23 (45.1)	15 (29.4)	0.115
Fertilization rate^a^	70.36 ± 16.9	74.11 ± 17.94	0.484	74.20 ± 13.81	74.34 ± 25.86	0.674	63.08 ± 16.34	78.69 ± 17.82	0.009	67.90 ± 16.41	74.57 ± 18.85	0.057
Implantation rate^b^	20.2 ± 18.7	14.5 ± 20.1	0.315	11.1 ± 16.6	11.1 ± 16.6	0.999	19.2 ± 19.0	17.7 ± 23.11	0.607	17.5 ± 18.7	13.6 ± 19.7	0.150
OHSS	3 (13.0)	1 (4.3)	0.317	1 (11.1)	0 (0.0)	0.317	0 (0.0)	1 (6.7)	0.317	4 (7.8)	2 (3.9)	0.687

*SD* standard deviation, *V* vaccinated, *I* infected, *r* pre, *s* post, *C* control, *T* total, *FHB* fetal heartbeat, *OHSS* ovarian hyper-stimulation syndrome.

^a^Fertilization rate is defined as the percentage of fertilized oocytes from the collected oocytes. ^b^Implantation rate is calculated as the number of gestational sacs observed per number of embryos transferred.

### COVID-19 vaccine

We found no evidence thatSARS-Cov-2 vaccination adversly affected fertilization rate, implantation rate, positive bHcg (OR 1.460, CI 0.735–2.901) and clinical pregnancy (positive fetal heartbeat) (OR 1.786, CI 0.886–3.603). Furthermore, IVF parameters did not differ significantly between those who were or were not vaccinated but the number of embryos at the cleavage stage was significantly lower in the vaccinated group (Table 3).

Similarly, mean values of the number of retrieved oocytes, fertilized oocytes, and number and class of embryos did not significantly differ in women before and after the vaccination (Table 4).

## Discussion

This study is consistent with already published results. As we found that, neither SARS-Cov-2 vaccine nor infection had a significant effect on IVF outcomes. Our results are in agreement with the currently available results of studies described in publications. Although the previously infected group had fewer embryos at the cleavage stage this didn’t affect the clinical outcome in terms of pregnancy. The latest study by Devora Aharon et al. in which they compared the IVF outcomes in 222 vaccinated patients versus 983 unvaccinated, were they found that there is no significant difference^38^. In addition to the previously mentioned study by Sarit Avraham et al. who came up with similar results^36^. Previously Bentov et al studied 32 IVF patients and found that follicular function was not altered by neither SARS-Cov-2 vaccine nor infection. Another study by Raoul Orvieto et al. among 36 patients came up with similar results^15,32^. Moreover, we couldn’t find any difference in the outcome between patients who received Sinopharm (BBIBP-CorV), Oxford-Astrazeneca or Pfizer(BioNTech) vaccine, in spite that the vaccines differ in their mechanism of action. Although, our sample size is small similar results was found in larger sample of over 100 women as mentioned earlier.

SARS-Cov-2 infection through the effect on angiotensin II can have a damaging effect on the ovaries and the endometrium. This infection can increase the circulating Angiotensin II due to reduction in ACE2 activity, so it can lead to changes in ovarian function, oocyte maturation and egg quality^39^. Moreover, Angiotensin II elevation may induce inflammation due to oxidative stress, consequently impairing reproductive ability^7^. TMPRSS4 is highly expressed in the endometrial cells in all phases of the menstrual cycle, especially during the window of implantation, furthermore, ACE2 expression increases in the mid secretory phase. Therefore, the secretory phase has a high risk of viral infectivity, although the evidence about the presence or absence of viral particles in the endometrial tissue is still lacking^11^. Accordingly, it could be anticipated that COVID-19 infection might has a harmful effect on female reproduction, and it has been proven by several studies that COVID-19 can detrimentally affect ovarian response and IVF outcomes^19–21^.

Furthermore, psychological stress due the pandemic might adversely affect reproductive system and fertility treatments outcome through its effect on the hypothalamic pituitary axis^40^. It has been hypothesized that stress increases reactive oxygen species (ROS) levels in the ovaries above acceptable physiological level, which may reduce the follicular growth. This appears to be more in infertile rather than the fertile women, though this hypothesis needs to be confirmed^41–43^. However, this can manifest itself during the peak of the pandemic, but further research is needed to investigate this effect.

Our study has several limitations, including relatively small sample size, and the retrospective design. Moreover. antibody levels were not assessed. However, what adds to the strength of our study is that we assessed the implantation rate, clinical pregnancy rate (using pregnancy test and positive fetal heart beat) which provides knowledge about the effect on early pregnancy. Moreover, we compared the outcome between different groups of IVF patients an addition to comparing the results in the same patients before and after the vaccination or infection. Further, more we looked at different types of vaccine.

Our study adds to the available evidence that COVID-19 vaccine is safe for patients planning to become pregnant. On the other hand, COVID-19 infection can have detrimental effects on a woman’s reproductive function. However, further research is needed to investigate the duration of the possible negative effect.

## Data Availability

The data underlying this article will be shared on reasonable request to the corresponding author.
